# What is the Source of Infections Causing Invasive Nontyphoidal *Salmonella* Disease?

**DOI:** 10.1093/ofid/ofad086

**Published:** 2023-02-20

**Authors:** Esmeda B Chirwa, Helen Dale, Melita A Gordon, Philip M Ashton

**Affiliations:** Institute of Infection, Veterinary and Ecological Sciences, University of Liverpool, Liverpool, United Kingdom; Salmonella group, Malawi Liverpool Wellcome Programme, Blantyre, Malawi; Institute of Infection, Veterinary and Ecological Sciences, University of Liverpool, Liverpool, United Kingdom; Salmonella group, Malawi Liverpool Wellcome Programme, Blantyre, Malawi; Institute of Infection, Veterinary and Ecological Sciences, University of Liverpool, Liverpool, United Kingdom; Salmonella group, Malawi Liverpool Wellcome Programme, Blantyre, Malawi; Institute of Infection, Veterinary and Ecological Sciences, University of Liverpool, Liverpool, United Kingdom; Salmonella group, Malawi Liverpool Wellcome Programme, Blantyre, Malawi

**Keywords:** iNTS, *Salmonella*, source, transmission

## Abstract

Invasive nontyphoidal *Salmonella* (iNTS) disease is a clinical condition distinct from *Salmonella* gastroenteritis. With an overall case-fatality rate of 14.5%, iNTS remains a major cause of morbidity and mortality, particularly in sub-Saharan Africa. However, the sources of infections that lead to cases of iNTS remain unclear. Broadly, there are 2 hypotheses as to the source of infections: (*i*) transmission from a zoonotic reservoir, similar to other nontyphoidal salmonelloses; or (*ii*) person-to-person transmission. Here we review several recent studies that have asked, “What is the source of infections causing invasive nontyphoidal *Salmonella* disease?” Two studies reported isolates in the stool of household members of iNTS cases that were very closely related (<3 single-nucleotide polymorphisms) to the iNTS case isolates; this is consistent with the hypothesis of person-to-person transmission, but infection from a common source (eg, a foodstuff) cannot be excluded. On the other hand, thorough investigations of the domestic environment of iNTS cases and the food pathway found only a single iNTS-associated *Salmonella* Enteritidis isolate. Therefore, we recommend that future studies test the hypothesis that iNTS is transmitted between people within the domestic environment. Further studies of food and water pathways are also warranted.

## Teaser

Invasive nontyphoidal *Salmonella* is a major cause of morbidity and mortality in Africa, but the source of infection remains unknown. This review of the available evidence suggests that person-to-person transmission is a more likely source than animal or environmental reservoirs.

Invasive nontyphoidal *Salmonella* (iNTS) is a significant cause of morbidity and mortality, primarily affecting sub-Saharan Africa (SSA). There were an estimated 535 000 (95% uncertainty interval, 409 000–705 000) cases, 77 500 (95% UI 46 400–123 000) deaths, and 4.26 million (95% UI 2.38–7.38) disability-adjusted life-years lost in 2017 [1]. Africa had the highest incidence rate of 51.0 cases (95% UI 36.3–68.0) per 100 000 persons per year and the highest case-fatality rate at 17.1% (95% UI 13.6%–21.0%) [2, 3].

**Figure 1.**
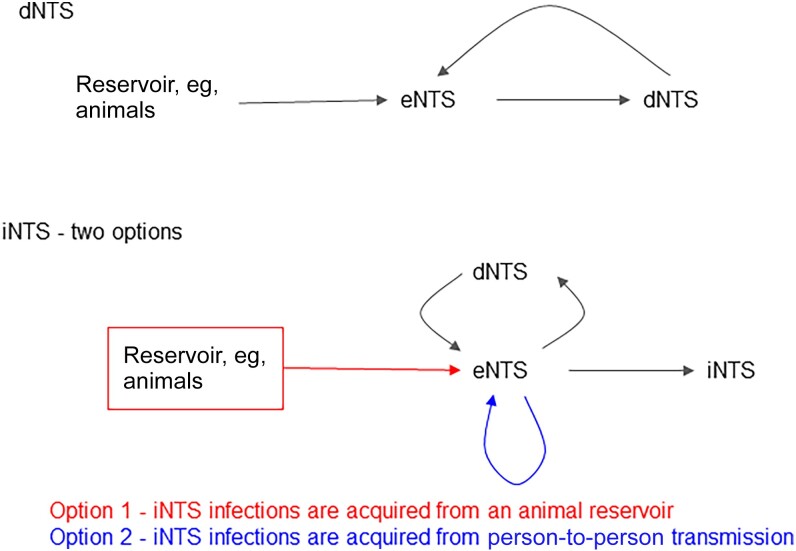
A schematic diagram of the known sources of diarrheal nontyphoidal *Salmonella* (dNTS) infection, and the 2 hypothesized sources of invasive nontyphoidal *Salmonella* (iNTS) infections. Option 1 for iNTS infections, highlighted in red, is that *Salmonella* from an animal reservoir colonizes the human gastrointestinal tract, leading to enteric nontyphoidal *Salmonella* (eNTS). Option 2 is that the source of infections leading to eNTS exposures are other humans with intestinal colonization of nontyphoidal *Salmonella*. In either case, once eNTS is established, there are 3 outcomes that can follow: (*i*) no active infection, just colonization; (*ii*) dNTS; or (*iii*) iNTS disease.

Most cases of diarrheal nontyphoidal *Salmonella* (dNTS) disease in high-income countries are zoonotic and foodborne. However, the source, or sources, of transmission for iNTS in SSA are unclear, with zoonotic, environmental, and human sources having been proposed (Figure 1). An improved understanding of sources of transmission of iNTS-associated strains could help in the design of interventions to reduce the burden of disease. For example, if a zoonotic reservoir were identified, then interventions such as animal vaccination or enhanced animal waste containment would be options that could reduce disease burden, whereas if person-to-person transmission were identified then development and deployment of a human vaccine would be an obvious priority.

In addition to iNTS and dNTS, we will use the term enteric nontyphoidal *Salmonella* (eNTS) to refer to asymptomatic colonization of the gut by a nontyphoidal *Salmonella* (NTS) isolate (see “Definitions” section for details). There is increasing evidence that humans can carry iNTS-associated strains in the gut without any symptoms (ie, as eNTS) [4, 5], and this is proposed to be a necessary condition for the development of invasive disease [6]. Yet conversely, most cases of iNTS are not associated with diarrhea [7], which emphasizes that dNTS and iNTS are clinically distinct phenotypes [8].

Most iNTS disease is caused by *Salmonella enterica* subspecies *enterica* serovars Typhimurium and Enteritidis (hereafter *S* Typhimurium and *S* Enteritidis), with particular subtypes being associated with invasive disease. For *S* Typhimurium, sequence type (ST) 313 has been frequently identified as the most common cause of iNTS disease, and where only multilocus sequence typing (MLST) data are available, we will refer to ST313 (and its single-locus variant ST3257) as iNTS-associated subtypes. As not all subclades of ST313 are associated with invasive disease [9], where whole genome sequencing (WGS) data are reported we will refer to the subclades of ST313 associated with invasive disease as iNTS-associated subclades. Similarly, for *S* Enteritidis, 2 subclades belonging to the same sequence type as the global diarrheal clade (ST11) have been described as being associated with iNTS [10], so where WGS data are reported, we will refer to the African *S* Enteritidis subclades as iNTS-associated subclades. As the identification of iNTS-associated subtypes/subclades requires molecular methods such as MLST or WGS, we generally only consider evidence from microbiological studies that use these methods. While bacterial traits that could lead to a higher invasive potential for these subtypes have been identified in both *S* Typhimurium and *S* Enteritidis, they have been more fully described for *S* Typhimurium, and they have been summarized in a recent review so will not be covered here [11].

**Figure 2.**
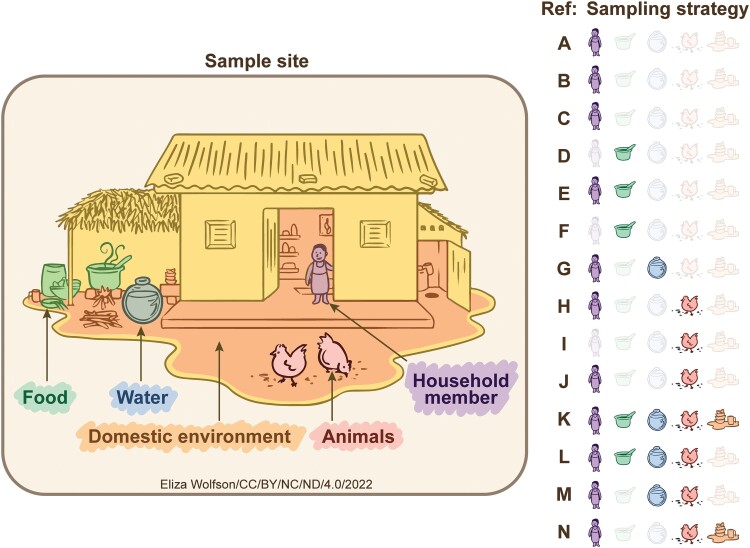
Household and environmental sampling strategies to identify the sources of nontyphoidal *Salmonella*. “Household members” refers to the index case or asymptomatic community control. “Food and food chain” refers to animal material from the point of animal slaughter to the point of being eaten. “Animal” refers to a live household-associated animal. “Water” refers to household stored water or nearby rivers/sewage/surface water. “Domestic environment” refers to the immediate household and surrounding surface environment. “Ref” refers to studies in Table 1.

Since early on in our understanding of iNTS, there has been uncertainty about the source and transmission of infections resulting in iNTS [12]. Here, we review the evidence addressing the question “what is the source of infections causing iNTS disease?” We have summarized the evidence by study type, grouped into the following categories: (*i*) comparisons of isolates from iNTS cases, their household members, animals, and environments; (*ii*) investigations of meat pathway, animal, and water source; (*iii*) associations between environmental factors and iNTS; and (*iv*) presence of iNTS-associated subtypes of NTS in diarrhea cases and controls. Table 1 and Figure 2 provide an overview of the evidence summarized in this review.

**Table 1.** Summary of the Evidence Presented in This Review

Sampling Strategy	Tested	Reference	Country of Study	Study Design	Sample Size	Key Finding Related to Sources of iNTS Transmission
**Humans (comparisons of invasive cases with asymptomatic, diarrheal, and hospital controls)**						
A	Humans	Kariuki et al, 2020 [4]	Kenya	Cases of iNTS and dNTS compared to eNTS from age-matched healthy children	53 iNTS and 30 dNTS compared to 50 eNTS from healthy children	WGS confirmed that the isolates from asymptomatic controls closely matched iNTS and dNTS cases
B	Humans	Kasumba et al, 2021 [5]	7 sites in Africa & Asia	Prospective, case-control study of the etiologic agents of moderate to severe diarrhea in children aged 0–59 mo	190 dNTS compared to 180 eNTS; 87 *S* Typhimurium were sequenced	*S* Typhimurium associated with invasive disease also causes diarrheal disease, and is also frequently isolated from asymptomatic people
C	Humans	Phoba et al, 2020 [6]	DRC	Case-control study of 1052 iNTS cases (blood and stool samples from same patient) compared with 1598 nonfebrile hospitalized controls	29% of iNTS blood isolates matched an isolate from their own stool, compared to 2.1% of afebrile controls having eNTS	High genetic similarity between NTS isolates obtained from the blood and stool samples of the same individual; afebrile humans as a potential reservoir of NTS
**Food and food chain**						
D	Food and food chain	Wilson et al, 2020 [19]	Kenya & Malawi	NTS isolated from fecal and mesenteric lymph of pigs at slaughterhouses	121 genomically confirmed NTS isolated	None of the NTS isolated from pigs belonged to the subclades associated with iNTS disease in Malawi, but were related to UK diarrheal strains
E	Humans, food and food chain	Crump et al, 2021 [18]	Tanzania & Kenya	Cattle, goat, and poultry meat pathway samples collected in Tanzania; human iNTS and dNTS samples collected in Kenya	164 NTS from meat samples, 90 dNTS from human samples, and 82 from iNTS	No evidence of *S* Typhimurium ST313 contamination of the food pathway. The meat pathway may be a source of human *S* Enteritidis infections
F	Food and food chain	Nikiema et al, 2021 [22]	Burkina Faso	Sandwiches from street food stalls in Ouagadougou were tested for *Salmonella*	*Salmonella* was isolated from 36/201 sandwiches	One *S* Enteritidis from an invasive lineage was isolated, no *S* Typhimurium isolated
**Water**						
G	Humans, water	Dekker et al, 2018 [21]	Ghana	iNTS isolates from hospitalized children compared with isolates from drinking water sources in their villages	165 NTS isolates from blood and 19 NTS isolates from water	No overlap of serotypes between humans and drinking water sources
**Animals**						
H	Humans, animals	Dione et al, 2011 [30]	The Gambia	dNTS cases and healthy community controls; rectal swabs taken from domestic animals of NTS cases	14 cases of dNTS and eNTS, 21 NTS from animal sources	No genomic evidence that HH animals in The Gambia act as a source of human infections
I	Animals	Carroll et al, 2021 [20]	South Africa	Retrospective analysis of NTS from livestock, companion animals, wildlife, and animal products over 60 y	63 NTS	No iNTS-associated subtypes were isolated from animal sources
J	Humans, animals	Falay et al, 2022 [23]	DRC	Isolates from rats trapped in marketplaces and a slaughterhouse were compared with isolates from iNTS cases in Kisangani, DRC	253 NTS from 46 rats; 40 *Salmonella* from human blood culture	ST313 lineage 2 was isolated from 3/566 (0.5%) rats captured; ST313 only isolated from systemic sites (liver & spleen), not from rat feces. Rat isolates clustered (cgMLST 5 allele threshold) with 5 of the 16 human iNTS ST313
**More than 2 sources: Humans, animals, environment, water, and/or food**						
K	Humans, water, food and food chain, animals, domestic environment	Kariuki et al, 2006 [12]	Kenya	iNTS compared to NTS in or near the case HH. Household sampling included rectal swabs from people, animals, food, water, environment (soil)	193 iNTS cases, 32 NTS from HH members, 10 NTS from animals and environment, typed by PFGE and antibiogram	Most NTS from the domestic environment that matched iNTS cases came from asymptomatic carriers, rather than environmental sources
L	Humans, animals, water, food and food chain	Kariuki et al, 2002 [13]	Kenya	Isolates of family members, food chain, water sources, and animal isolates in or near the HHs of iNTS or dNTS cases	151 NTS from humans, 78 from animals, water, or food by PFGE and antibiogram	There was not a close relationship between iNTS isolates and NTS from animals living in close contact
M	Humans, animals, water	Post et al, 2019 [14]	Burkina Faso	iNTS compared with NTS from their HHs. Sampling of rectal swabs from family members and animals, and drinking water	32 index patients, 16 NTS isolates from livestock, 18 NTS from HH members	WGS showed the only HH isolates that matched iNTS cases were from asymptomatic family members
N	Humans, animals, domestic environment	Koolman et al, 2022 [17]	Malawi	iNTS strains compared with NTS from the index HH and geographically matched HHs. Sampling of stool/rectal swabs from family members and domestic animals, and boot-sock sampling of the HH environment	28 iNTS cases, 43 NTS from case and control HHs	WGS showed the only HH isolates that were closely related iNTS cases were case-matched family members

Abbreviations: cgMLST, core genome multilocus sequence type; dNTS, diarrheal nontyphoidal *Salmonella*; DRC, Democratic Republic of the Congo; eNTS, enteric nontyphoidal *Salmonella*; HH, household; iNTS, invasive nontyphoidal *Salmonella*; NTS, nontyphoidal *Salmonella*; PFGE, pulsed-field gel electrophoresis; ST, sequence type; UK, United Kingdom; WGS, whole genome sequencing.

## DEFINITIONS

- Enteric nontyphoidal *Salmonella* (eNTS): Where the gastrointestinal tract is asymptomatically colonized by nontyphoidal *Salmonella*. It can either precede or follow *Salmonella*-induced illness, or not be associated with illness at all.
- Diarrheal nontyphoidal *Salmonella* (dNTS): Refers to diarrheal symptoms with NTS isolated from the stool.
- Invasive nontyphoidal *Salmonella* (iNTS): Where symptomatic disease occurs (eg, sepsis, meningitis) with NTS isolated from a usually sterile site (ie, bloodstream or cerebrospinal fluid).

## COMPARISON OF ISOLATES FROM iNTS CASES WITH ASYMPTOMATIC CARRIERS AND ANIMAL AND ENVIRONMENTAL SOURCES

A pioneering study in Nairobi, Kenya, published in 2002, compared pulsed-field gel electrophoresis (PFGE) profiles and antibiogram patterns of isolates causing human iNTS and dNTS isolates with those obtained from animals, food, or the environment [13]. The PFGE patterns of both *S* Enteritidis and *S* Typhimurium isolates from humans were distinct from those from animal or environmental sources. A follow-on study published in 2006 compared NTS isolates from children with iNTS disease admitted at 2 hospitals in Nairobi, Kenya, with NTS serotypes and genotypes isolated from family contacts and environmental samples from the index cases’ homes [12]. They found that the antibiotic susceptibility profiles and plasmid content of 21 of the 32 (65.6%) NTS acquired from index cases’ contacts (9 adults and 23 children) were identical and their PFGE patterns indistinguishable from those of the index cases. In contrast, the isolates from livestock and environmental samples differed from those found in index cases (ie, only 3 of 180 environmental samples matched those of index cases). *Salmonella* Typhimurium and *S* Enteritidis were isolated from contacts of index cases in similar proportions to those found in cases, implying that human-to-human transmission could be relevant for both serotypes.

To explore the transmission and reservoir of *Salmonella* causing iNTS in Burkina Faso, Post et al performed a blood culture surveillance study among children <15 years (index cases) [14]. A household survey was performed for each index case, during which drinking water, household members’ stool, and livestock stool were collected and cultured for *Salmonella*. Isolates from index cases’ blood cultures were compared to isolates from their respective households using multilocus variable-number tandem-repeat analysis (MLVA) and WGS. Of the 32 index cases included in this study, 26 were *S* Typhimurium, 5 were *S* Enteritidis, and 1 was *Salmonella* Freetown. All *S* Typhimurium isolates were ST313. *Salmonella* Typhimurium and *S* Enteritidis were not found in any of the livestock samples. In 3 households, Post et al identified *S* Typhimurium ST313 in both the index patient’s stool sample and the stool sample of a household member, with 0–2 single-nucleotide polymorphism (SNP) difference between pairs in the same household. There were no matching serotypes between index cases and livestock or water samples from their households. Although their sample size was a limitation, Post et al interpreted their findings as providing evidence of a human reservoir for iNTS in SSA. In another study in the Democratic Republic of Congo, Mbuyi-Kalonji et al compared the MLVA types of 4 NTS isolates from people without iNTS with those from 19 iNTS cases, and found that the 3 out of 4 non-iNTS isolates were identical to an iNTS isolate [15].

In 2020, Kariuki et al published a case-control study over a 5-year period in informal urban settlements in Nairobi, Kenya, where they compared iNTS and dNTS disease–causing isolates from children aged <16 years with asymptomatic carriage isolates (eNTS) among age-matched controls [4]. The only 2 *S enterica* serotypes associated with invasive disease in this study were *S* Typhimurium (52.5%) and *S* Enteritidis (47.5%). Phylogenetic analysis of the WGS data demonstrated a high level of similarity between NTS isolates obtained from all 3 clinical phenotypes (iNTS, dNTS, eNTS), indicative of infection from a common source pool. Isolates from individual cases and controls exhibited strong genetic relatedness, with some cases and controls having genotypes that were 0 core genome SNPs difference. They also observed a significant rate of asymptomatic carriage (0.79%) of iNTS-associated subclades in the population that could serve as a potential source of community transmission. Msefula et al found that invasive *S* Typhimurium isolates from Malawian children and human immunodeficiency virus (HIV)–infected adults were highly genetically related, with no clustering of strains by HIV status or age, implying that these isolates are freely transferred between these populations or were acquired from a common source [16].

Koolman et al undertook extensive human, animal, and domestic environment sampling of iNTS and typhoid index-case households and geographically matched control households in Blantyre, Malawi [17]. They combined extensive boot-sock environmental sampling with human and animal sampling, as well as WGS analysis of the resulting isolates, to determine the relatedness of invasive index strains with NTS from household sources. Household isolates were all NTS and yielded 15 different STs. In contrast, iNTS human disease was only caused by 3 STs of *S* Typhimurium, primarily ST313. Despite yielding a high number of NTS serotypes from household samples, there was no overlap between STs causing iNTS disease and NTS from animal or environmental samples. The only sample type where they found *Salmonella* that matched strains causing iNTS disease was from asymptomatic household members of the index cases (2 and 3 SNPs difference, respectively). These findings suggest person-to-person transmission, or infection from a common source, and are consistent with the hypothesis that humans are the primary source of the organisms responsible for iNTS disease in SSA.

Although these studies found no genetic similarity between animal or environmental NTS and NTS causing invasive disease, they did find genetic similarity between iNTS causing isolates and eNTS from asymptomatic household members. Asymptomatic household members with eNTS could therefore be a source for the transmission of iNTS-associated subtypes.

## MEAT PATHWAY, ANIMAL, AND WATER SOURCE STUDIES

Another way to look at potential sources or reservoirs of iNTS infection is at a food-systems level, for example, along the meat pathway, including animals raised for food. If iNTS-associated subtypes or subclades were identified in these potential sources or reservoirs, that would provide evidence of the potential for those food system components to act as a source of iNTS infections.

Crump and colleagues tested for NTS in cattle, goat, and poultry meat pathway samples in Tanzania [18]. They isolated 164 NTS from the meat pathway samples, including from poultry farms (n = 33), ruminant slaughter/butcher environments (n = 32), cattle or their meat (n = 62), and goats or their meat (n = 37). They used WGS to compare the meat pathway NTS isolates with 172 NTS from humans in Kenya, including 90 (52%) from stool and 82 (48%) from blood. Despite the high yield of NTS from the meat pathway samples, the only place they isolated ST313 from was human samples. They observed core genome MLST clusters of *S* Enteritidis ST11, *Salmonella* Heidelberg ST15, *S* Typhimurium ST19, and *Salmonella* II 42:r:- ST1208 that included both human and meat pathway isolates, indicating that they were successful at identifying zoonotic clades. They concluded that the existence of a nonhuman reservoir for *S* Typhimurium ST313, if any, remains to be established. Their *S* Enteritidis ST11 isolates formed core genome MLST clusters with the global epidemic clade, rather than the invasive associated African clades.

Wilson et al cultured NTS from the feces and mesenteric lymph nodes of pigs at slaughter from 2 sites in Kenya and 1 site in Malawi [19]. They observed NTS carriage rates of 12.7% and 9.1% from the 2 sites in Kenya (Nairobi and Busia), and 24.6% in Malawi (Chikwawa). They isolated a total of 149 NTS, including 61 from Busia, 40 from Nairobi, and 20 from Chikwawa. WGS revealed 32 different serovars from 2 subspecies of *Salmonella*: *S enterica* subspecies *enterica* and *S enterica* subspecies *salamae.* Eight isolates of *S* Typhimurium were identified, with 6 belonging to ST19 and 2 belonging to ST313. However, the 2 ST313 isolates, from pigs in Kenya, did not belong to the subclades of ST313 that have been associated with invasive disease in Africa, but rather subclades associated with gastroenteritis in England and Wales [9]. The authors concluded that their data revealed the potential for zoonotic transmission of diarrheal *Salmonella* strains to humans, but not for the transmission of clades associated with iNTS. This finding of isolates belonging to gastroenteritis-associated subclades of ST313 in the food pathway in Malawi is remarkable. The fact that “UK-ST313” is present in the food system in Kenya, but has never been associated with invasive disease, strengthens the idea that invasive lineages of ST313 have specific biological adaptations facilitating systemic dissemination.

Carroll et al sequenced 63 isolates from companion, wild, and domestic animals, and animal products from South Africa over a 60-year period [20]. In total, there were 26 *S* Typhimurium isolates, of which 9 were isolated from 2005 onward. No ST313 were seen in this study. This study is impressive in its historical collection, but South Africa does not have a high burden of iNTS disease, and most of the isolates from the study were before the iNTS epidemic peaked, so the results as they relate to the question of iNTS-associated types should be interpreted with caution. Dekker et al investigated the potential for water sources to be linked to iNTS cases in rural Ghana; they compared 165 iNTS cases with 19 NTS from drinking water sources in the same community and found no overlap of serotypes between the 2 niches [21].

Nikiema et al investigated the contamination of street food sandwiches with *Salmonella* in Ouagadougou, Burkina Faso [22]. The prevalence of *Salmonella* was 17.9%, with 16 different serotypes isolated, with the most frequent being Kentucky, Derby, and Tennessee. One multidrug-resistant *S* Enteritidis belonging to an invasive clade (West African subclade) was isolated from a beef kebab sandwich. This finding is the first finding of an iNTS-associated subclade being identified in a food source.

Falay et al investigated the hypothesis that rats in an urban environment could carry iNTS-associated subtypes [23]. They captured 566 live rats from markets and a slaughterhouse in Kisangani, Democratic Republic of the Congo. They isolated 253 NTS from 46 rats (8.1% prevalence), including 12 *S* Typhimurium from 10 rats; no *S* Enteritidis was found. Core genome MLST revealed that 4 isolates from 3 rats (3/566 [0.5%]) belonged to ST313 lineage 2 (which is associated with invasive disease). Furthermore, on comparison with 16 genome sequences of *S* Typhimurium ST313 lineage 2 from invasive human disease, they found that 5 of 16 human iNTS *S* Typhimurium were part of 5-allele-difference core genome MLST clusters with isolates from rats, indicating potential epidemiological connections between the rats and human invasive disease. However, they only isolated ST313 from deep organs (liver and spleen) of rats, not from feces. The authors highlighted that more fieldwork should be done to identify if this finding is repeated elsewhere, and also laboratory work on the pathophysiology of iNTS in rats (eg, fecal shedding).

Only 1 of these studies above found any evidence that iNTS-associated subclades are present in food systems, and 1 study identified iNTS-associated subclades from rats. These intriguing findings should be set against the 3 other studies that found no evidence of iNTS-associated subclades in food or animals. No study identified iNTS-associated subclades from water sources. Due to the small number of studies on this subject, and the intriguing findings of some of them, we strongly recommend that more studies of this kind be carried out, with further considerations to strengthen the study design. This may include a more systematic “farm to fork” approach with extensive sampling from people, animals, and meat along the pathway from farms/site of livestock rearing and surrounding environment, slaughterhouses, storage facilities, butchers, villages, and individual households. Rats should certainly be sampled in future studies. This could be supported by detailed food surveys of iNTS cases, household members, and local villages, as well as a better understanding of the food pathways that exist in endemic settings where livestock rearing, slaughtering, storage, and consumption may happen in close geographical proximity or may encompass wide geographical areas.

## ASSOCIATION BETWEEN ENVIRONMENTAL FACTORS AND iNTS INCIDENCE

Seasonal variation in infectious disease burden is often a sign that there is an environmental role in the epidemiology of the disease. iNTS has been shown to be seasonal, with the peak of disease occurring during and after the rainy season [24, 25]. However, this effect has been thought to be largely indirect, due to the seasonal nature of iNTS risk factors such as malaria and malnutrition [26]. Malaria incidence increases during the rainy season because of more water sources for mosquitoes to breed in, while acute malnutrition increases during the “hunger season”—shortly before crops are harvested, or through droughts and flooding impacting crop harvests [27]. Therefore, it is key when estimating the impacts of climate factors on iNTS that the seasonality of risk factors is also considered.

A study set in urban Blantyre, Malawi, with low malaria and high HIV prevalence, used structural equation models to investigate relationships between longitudinal iNTS blood culture positivity, malaria slide positivity in febrile children, pediatric HIV prevalence, malnutrition ward admissions, and monthly rainfall over 9 years [28]. Malaria and malnutrition were found to have statistically significant and direct contributions to iNTS disease; however, rainfall was found to only have an indirect effect through its effect on malaria and malnutrition.

A study in the Democratic Republic of Congo, in a setting with high malaria and malnutrition prevalence, concluded there was a direct relationship between the seasonal patterns seen in iNTS incidence, outside of the impact of seasonality of host risk factors [29]. Principal component analysis was used to separate the effect of rainfall and temperature from host factors measured by monthly malaria slide positivity, pediatric blood transfusions, and malnutrition admissions into independent components, followed by time series regression to assess each component's independent association with iNTS incidence seasonality. They concluded that host factors alone accounted for 17.5% and rainfall an independent additional 9% of the monthly variation of iNTS observed—indicating a direct impact of rainfall on iNTS seasonality independent of seasonal variation in host susceptibility. Temperature showed an initial association with iNTS seasonality, but lost significance in the multivariate model, and no delayed effect of environmental factors on iNTS seasonality was found.

In summary, these studies only indirectly address the question of environmental transmission of NTS—using statistical models in an attempt to unpick the relationships between seasonality in host susceptibility and seasonal climatic factors. The association between rainfall and iNTS incidence could indicate an environmental reservoir such as animal fecal material, from which dispersal is enhanced with the onset of the rainy season. However, enhanced transmission during the rainy season could be associated with contamination of unimproved water sources with human faeces, which has been repeatedly shown to contain iNTS-associated subtypes. Despite this ambiguity, identifying the direct effects of climatic factors on iNTS disease does leave open the possibility of a nonhuman reservoir, whereas an indirect association mediated through known risk factors (eg, malaria, malnutrition), would argue against an environmental source.

## NTS FROM DIARRHEA CASES AND CONTROLS

If subtypes of *Salmonella* that are associated with iNTS are found to frequently cause diarrhea, or be present in the stool of healthy people, then it increases the plausibility of the person-to-person route of transmission via the fecal–oral route. Furthermore, studies on the environmental sources of diarrhea-causing isolates can provide information on possible routes of transmission of *Salmonella*.

In 2011 Dione and coworkers reported a study of children in rural Gambia that investigated the role of domestic animals in the transmission of NTS to humans [30]. They carried out active, population-based, case-control surveillance of severe diarrhea as part of the Global Enteric Multicentre Study (GEMS). Fieldworkers visited the homes of any children (either diarrhea cases or healthy) who were infected with NTS and took rectal swabs from domestic animals, including goats, sheep, and chickens. The NTS isolates were typed using MLST. There were 14 children who were positive for NTS, and a total of 210 domestic animals were sampled. NTS was identified in 21 of 210 animal samples, with 6 of 14 households positive for NTS in at least 1 animal sample. There was no *S* Typhimurium or *S* Enteritidis isolated from either human or animal sources. There was no overlap between the serotypes observed in animals and the serotypes observed in people. The authors concluded that their findings did not support the hypothesis that humans and animals in close contact in the same household carry genotypically similar *Salmonella* serovars. Feasey et al compared *S* Enteritidis from multiple sources in SSA, including isolates from invasive and noninvasive body sites [10]. While they reported the association of 2 African-associated subclades with invasive disease, they also identified Enteritidis belonging to these subclades in samples from noninvasive body sites (eg, stool).

GEMS enrolled children aged 0–59 months with moderate to severe diarrhea from 4 sites in Africa and 3 sites in Asia. Kasumba and colleagues built on top of GEMS by analyzing nontyphoidal *Salmonella* from the stool of both cases and controls [5]. They identified 87 *S* Typhimurium from cases of diarrhea and healthy controls from Kenya, Pakistan, India, and Bangladesh [5]. They found that 50 of 55 (91%) sequenced isolates from diarrheal or healthy children belonged to the ST313 lineage 2 subclade associated with iNTS disease. This report is also notable for being the first analysis to demonstrate that ST313 is associated with diarrhea in a case-control study. The authors concluded that widespread asymptomatic and diarrheal carriage of iNTS-associated subclades supports the hypothesis of person-to-person transmission. Akullian et al compared isolates of NTS from cases of iNTS and cases of diarrhea in western Kenya. *Salmonella* Typhimurium ST313 was observed to cause both invasive and diarrheal disease [31].

In summary, iNTS-associated subtypes and subclades can be frequently isolated from the stool of both children with diarrhea and healthy children. This increases the plausibility of the hypothesis of person-to-person transmission via a fecal–oral route.

## CONCLUSIONS

There is good evidence now from multiple studies in multiple settings that the source of iNTS-associated types is not the domestic environment (neither physical nor animal). Two studies have found isolates that match iNTS index cases in the stool of household members of those iNTS cases. Further work needs to be done to confirm the hypothesis that iNTS-associated lineages can be transmitted between people living in the same household. The available studies that have taken a systematic approach to sample animal reservoirs, food pathways, or water sources have rarely (1 isolate from 1 study) found that these could act as a source of iNTS-associated subtype/subclade infections. More investigations of food pathways and water sources should be carried out at a systemic level, as targeted investigation of the food consumed by iNTS cases is very challenging. While MLST is sufficient to identify ST313, which has been associated with iNTS, we know that not all ST313 have been associated with iNTS, and that non-iNTS-associated subclades of ST313 have been isolated from animal sources in Kenya. Therefore, we recommend WGS and full phylogenetic analysis for future projects.

## Notes

***Disclaimer.*** The funders had no role in study design, data collection and analysis, preparation of the manuscript, or decision to submit the manuscript for publication.

***Financial support.*** The Malawi Liverpool Wellcome Programme is core-funded by a grant from Wellcome (206545/Z/17/Z). M. A. G. is supported by a Research Professorship from the UK Department of Health, National Institute for Health Research (NIHR300039). Philip Ashton and Esmeda Chirwa are both supported by Professor Melita Gordon's NIHR research Professorship. Wellcome PhD Training Fellowship for Clinician awarded to Helen Dale (222931/Z/21/Z).
